# Boys and girls

**DOI:** 10.1186/2041-2223-3-13

**Published:** 2012-06-20

**Authors:** Mark A Jobling

**Affiliations:** 1 Department of Genetics, University of Leicester, University Road, Leicester, LE1 7RH, UK

## Sex matters. The first question anyone ever asked about you was probably *Boy or girl?*, and thanks to the X/Y sperm lottery, the odds were pretty much even. Sex ratios at birth in species that use chromosomal sex-determining systems are generally close to 1:1. In humans, there’s a slight male excess (a male: female ratio of 1.05 on average), a fact first noted in a paper published over 300 years ago [1], with the charming title: *An argument for divine providence, taken from the constant regularity observ'd in the births of both sexes*. Because differential sex mortality favours females, as time goes on the ratio evens itself out to 0.98 – 1.00, a figure recorded in the census data of many western countries.

These days, nature’s big surprise is frequently spoilt by the intervention of a medical sonographer. Reading the *Observer* newspaper a few weeks ago, I came across an article [2] about baby gender-reveal parties. The parents-to-be cut into a large cake, and thus reveal the sex of the baby to the assembled masses by the pink or blue sponge hidden beneath the icing. Some examples have the message *He? She? Open to see!* iced on the top. In the most extreme cases, the ultrasound scan is delivered direct to the baker in a sealed envelope, so that even the parents themselves don’t know. If you’re really interested, YouTube has the video evidence.

This cutesy practice is all very well, but of course there’s a darker side to knowing the sex of a baby, and female foeticide in societies where male babies are more highly valued has become commonplace since the 1980s, thanks to the availability of ultrasound scanning. Abortion on the grounds of sex is illegal, but it goes on nonetheless. Interestingly, the gender-reveal story described above was accompanied, in the same issue, by a small advert featuring the beatific face of an unborn baby, with the words *BARGAIN! Pre-used Ultrasound Scanners at a remarkable price!* plus an email address and phone number. Now, I wonder who might want such things?

Sex-ratios in some societies have shifted alarmingly from the norm. For example, in parts of India (Delhi, Gujarat and the Punjab), male: female ratios for young children are between 1.14 and 1.26, while in some rural provinces of China they reach 1.30 [3]. The clear influence of sex-selective abortion is seen in sex-ratios at birth for later children - in South Korea, the ratio for fourth births is an amazing 2.29 – surely no lottery there. The problem in China is exacerbated by the so-called ‘one child’ policy, and by a variant that permits a second child if the first is a girl. There is considerable alarm about the future social consequences of the excess of males in some societies.

Prior to the ultrasound era, adjusting the sex ratio was a different business, as illustrated by a 2001 paper in *Forensic Science International*[4] disturbingly entitled, *Traffic injury or attempted infanticide?* It tells the tale of a young woman in Wuhan, in Central China, struck by a lorry while cycling. She falls from her bike into a pile of rubbish from a clothing factory and, unconscious, is admitted to hospital and examined. Her scalp is lacerated and the X-ray reveals a neck fracture, but also something more unexpected – two large sewing needles (their ‘eyes’ clearly visible in the images) in her brain. Her husband takes the not unreasonable view that they must have arrived there via the fall into the garment waste, but there is a problem – their angles are very different, and they lie beneath the bone of the skull. The conclusion? – they were separately placed there before her fontanelles were closed, so someone tried to kill her when she was less than two years old. The author points out that female infanticide is not unusual in China, and the method inferred here is ‘an easy and secret way to kill the unwanted baby’. There are several other similar reports in the literature.

All this distressing foeticide and infanticide is in aid of a small and intronless gene on the short arm of the Y chromosome, *SRY*, whose product acts to trigger the differentiation of the testis [5]. The acronym denotes a monumentally useless gene-name, *sex-determining region, Y*, reflecting the paranoia of the gene-hunters back in the late 1980s. The previous best candidate gene (accompanied in the *Cell* paper by allusions to Aristotle and his *De Generatione Animalium*[6]) had proven to be a red herring, so when a better candidate was found, its discoverers hedged their bets. The name (how can a ‘region’ be a ‘gene’?) has served to confuse students ever since. It’s only recently that we’ve come to understand how *SRY* actually works: the protein acts on the enhancer of the *SOX9* gene to trigger the differentiation of Sertoli cells [7], which have key roles in repressing female-specific structures early on, and in spermatogenesis later.

Given the fundamental nature of sex in the lives of almost all higher organisms, it’s astonishing what an exotic gallery of independent evolutionary novelties it involves. We can see bewildering new inventions emerging among rodents [8]: some species of *Ellobius* mole voles have jettisoned *SRY* and the Y chromosome, males and females having identical XX karyotypes; some Japanese spiny rats (*Tokudaia* species) have done the same, but show odd-numbered ‘XO’ karyotypes. In the creeping vole (*Microtus oregoni*) mosaicism is involved, with the females being XO in somatic cells, but XX in the germ-line, and the males XY in the soma, but losing the X chromosome altogether in the germ cells. It’s not yet known how the sex-determination process works in these enigmatic creatures.

Beyond the mammals, there are more exotica still. In birds, it’s the eggs (from ZW females), rather than the sperm (from ZZ males), that decide the sex of the chick. In the fruit-fly *Drosophila melanogaster*, there’s a Y chromosome, but it has nothing at all to do with sex determination, which is instead determined by an elegant mechanism for measuring the ratio of X chromosomes to autosomes. In many reptiles, chromosomes are irrelevant, the sex of offspring being determined by the temperature at which the embryos develop. This led to the theory that dinosaur extinction might have been triggered by a sudden dramatic elevation in temperature, possibly triggered by the Chicxulub asteroid impact in the Yucatán peninsula, and leading to a disastrously unisex population [9].

Imagine a species that uses temperature-dependent sex-determination, but has invented the thermostat: thank goodness we don't do it that way.
